# In Vitro Evaluation of the Antiviral Effect of *Spirulina maxima* (*Arthrospira*) Alga Against Chikungunya Virus

**DOI:** 10.3390/v17121583

**Published:** 2025-12-05

**Authors:** José Angel Santiago-Cruz, Araceli Posadas-Mondragón, José Leopoldo Aguilar-Faisal, Cesar Ismael Ortiz-García, Danai Montalvan-Sorrosa, Norma Estela Herrera-González, Angélica Pérez-Juárez

**Affiliations:** 1 Laboratorio de Medicina de Conservación de la Sección de Estudios de Posgrado e Investigación, Instituto Politécnico Nacional, Escuela Superior de Medicina, Plan de San Luis, Colonia Casco de Santo Tomas, Ciudad de México 11340, Mexico; jsantiagoc@ipn.mx (J.A.S.-C.); jaguilar@ipn.mx (J.L.A.-F.); 2 Laboratorio de Genética Microbiana, Instituto Politécnico Nacional, Escuela Nacional de Ciencias Biológicas, Manuel Carpio, Colonia Casco de Santo Tomas, Ciudad de México 11340, Mexico; cortiz@ipn.mx; 3 Departamento de Biología Celular, Facultad de Ciencias, Universidad Nacional Autónoma de México, Circuito Exterior Ciudad Universitaria, Alcaldía Coyoacán, Ciudad de México 04510, Mexico; danai@ciencias.unam.mx; 4 Laboratorio de Oncología Molecular de la Sección de Estudios de Posgrado e Investigación, Instituto Politécnico Nacional, Escuela Superior de Medicina, Plan de San Luis, Colonia Casco de Santo Tomas, Ciudad de México 11340, Mexico; nherrerag@ipn.mx

**Keywords:** *Spirulina maxima* (*Arthospira*), Chikungunya, cytotoxicity, antiviral

## Abstract

Chikungunya fever (CHIKV) has reemerged as a serious global health problem worldwide. Currently, no antiviral drugs are available for the prevention or treatment of CHIKV infection. Therefore, this study aimed to analyze the anti-CHIKV potential of the alga *Spirulina maxima*. Extracts were obtained by maceration using solvents of different polarities (hexane, dichloromethane, and methanol). The cytotoxic activity of the extracts was evaluated using the MTT assay, which demonstrated that none of the extracts had a cytotoxic effect on VERO and BJ cell lines. The anti-CHIKV effect was evaluated using a plate assay with VERO, HepG2, and BJ cell lines. The results showed that the methanol extract had the best anti-CHIKV effect, completely inhibiting viral replication at a concentration of 15 µg/mL in all three cell lines. Furthermore, the addition time assay suggested that the mechanism of action is related to the inhibition of some processes at the beginning of the infection, such as the entry or early replication of the virus. In conclusion, SP contains molecules that could provide a basis for future research on the development of new drug therapies against CHIKV.

## 1. Introduction

Chikungunya virus (CHIKV) is an arthropod-borne virus that belongs to the genus *Alphavirus*, family *Togaviridae* [1]. It is transmitted by infected *Aedes* spp. mosquitoes, with *Aedes aegypti* being the main vector of transmission [2]. Since this virus was first isolated in Tanzania in 1952, it has spread throughout Africa, Asia, Europe, and America, where multiple outbreaks of fever caused by this virus have occurred in more than 50 countries [3]. CHIKV infections have recently reemerged, with a pandemic reported in 2015 on the American continent. It should be noted that the increases in the number of cases and deaths from CHIKV are greater than those reported in recent years and are added to the simultaneous circulation of other arboviruses [4]. Currently, this virus is circulating in Africa, Asia, and the Americas. By March 2025, approximately 80,000 cases and 46 deaths from this virus were reported in 14 countries. In America, the countries that report the highest number of cases are Brazil (71,578), Argentina (1550), Bolivia (77), and Peru (32) [5].

The acute clinical presentation of the disease caused by CHIKV has a manifestation of symptoms that are not fatal for the patient, but considerably compromise their quality of life since the symptoms include high fever (≥39 °C), arthralgia which manifests itself intensely in wrists, hands, knees, and feet [6,7], muscle pain (myalgia), rash, fatigue, headache, and in some cases, affect the central nervous system, causing optic neuropathy, neuroretinitis, and encephalitis, with the highest mortality rate occurring in these latter cases [8,9]. These symptoms can persist for weeks, months, or even years in chronic cases.

One of the most worrying aspects is that the treatment given to patients only focuses on alleviating the symptoms of the disease [10]; therefore, in this and other arboviral diseases, there are no antivirals available [11] to prevent these infections or fight the virus during infection, preventing its spread in the various cells of patients and avoiding the excessive damage that these viruses, such as CHIKV, can cause.

This virus has great potential to cause a pandemic and has been placed on the list of priority pathogens for vaccine development [12,13]. This is why multiple efforts have been made in the research of different therapeutic protocols to identify possible antiviral candidates against CHIKV infection [14,15,16]. One approach is based on molecules obtained from natural products with antiviral activity in vitro and in vivo, which have a potential effect against CHIKV infection [17,18].

Microorganisms are a source of molecules obtained from natural products, and many of them have been used to obtain molecules with antiviral effects. For example, actinomycetes are one of the main producers of antiviral molecules [19,20]. Cyanobacteria and microalgae are another large group of microorganisms from which molecules with antiviral activity against human viruses have been isolated. These microorganisms produce extracellular polymers and polysaccharides, such as α-glucans and β-glucans, which have shown antiviral effects against HIV-1, herpes simplex virus type 1, SARS-CoV-2, Hepatitis C Virus (HCV), influenza A and B viruses, Japanese encephalitis virus (JEV), and white spot syndrome virus (WSSV) [21,22,23].

The cyanobacterium *Spirulina maxima* (SP), also known as blue-green algae, contains a high level of nutrients such as protein, carbohydrate, lipopeptides, essential fatty acids, amino acids, minerals, vitamins, and photosynthetic pigments, and has been used worldwide as a food supplement [24]. In addition to its high nutrient content, other beneficial properties for humans have been observed, particularly in the medical field, as the molecules present in SP have various biological activities, including antioxidant, antifungal, antibacterial, antitumor, and immunomodulatory activities [25,26,27].

Some of the molecules are isolated from this cyanobacterium exhibit antiviral activity. SP has been used as an alternative natural therapeutic agent for different viral infections, including herpes simplex type 1 (HSV-1), mumps virus, COVID-19, influenza A virus (IAV), and cytomegalovirus, due to its wide content of bioactive compounds, mostly its pigments, such as allophycocyanin, fragments of polysaccharides, fatty acids (sulfolipids), minerals, and other components that have presented antiviral properties [28,29,30,31].

This study aimed to determine, for the first time, the anti-CHIKV potential of metabolites present in a commercial formulation (SDW-9714) of SP. We found that the methanolic extract contains molecules capable of completely inhibiting chikungunya virus replication in vitro in three different cell lines, and that the concentrations necessary to achieve this effect do not cause cytotoxicity. In addition, we determined that the possible mechanism of action may be involved in the inhibition of viral genome replication.

## 2. Materials and Methods

### 2.1. Spirulina maxima (Arthospira) Used in the Tests

We evaluated the anti-CHIKV potential of a food supplement developed by Productos Esenciales para la Humanidad, S.A. de C.V., CDMX, Mexico. It consists of the dry biomass of *Spirulina maxima* (SP). Bulk production batch SDW-9714 was used in this study.

### 2.2. Preparation of Spirulina maxima (Arthospira) Extracts

To determine the anti-CHIKV activity of the commercial SP powder, 0.001 g of the powder was weighed and dissolved in 100 µL of HPLC-grade water (Millipore Sigma, MWX0008-1, Burlington, MA, USA), obtaining a suspension called SP-W at a concentration of 1000 µg/100 µL.

After demonstrating the anti-CHIKV activity in SP, we prepared extracts using solvents of varying polarities to purify the compounds of interest. SP (50 g) was weighed and placed in a glass container, and 250 mL of hexane was added. This mixture was allowed to stand for 3 days, and the solvent was subsequently filtered and evaporated using a rotary evaporator (IKA-0010003491, Wilmington, DE, USA). The solid residues were subjected to extraction with hexane on two more occasions; all the solvent was evaporated, and thus the hexane extract (SP-H) (6 g) was obtained. The solid residues were left to dry in the shade and then subjected to the same procedure with dichloromethane, thus obtaining the extract with this solvent (SP-D, 18 g), and subsequently, the methanolic extract (SP-M, 11 g) was obtained using the same procedure. All solvents were of analytical grade and purchased from Arben Chemistry (Mexico State, Mexico). The solvents were distilled before use.

### 2.3. Chemical Composition of SP Methanol Extract (SP-M)

SP-M was subjected to MS/MS analysis. LC-MS analysis was performed using a Vanquish Flex UHPLC system coupled to an Orbitrap Exploris 120 mass spectrometer (ThermoScientific, Waltham, MA, USA, EE. UU). The column used was Kinetex XB-C18 100A 150 mm × 2.1 mm (Phenomenex, Torrance, CA, USA). SP-M (1 mg) was dissolved in 500 µL of dichloromethane and diluted 10:100 in 0.1% formic acid in methanol (Sigma Aldrich, Mexico State, Mexico; high-performance liquid chromatography grade). Chromatographic separation was performed using mobile phases A (0.1% formic acid) and B (0.1% formic acid in methanol). The separation was carried out using the following gradient: 0–2 min, 10% phase B; 2–20 min, 10–100% phase B; 20–27 min, 100% phase B; and 27–28 min, 100–10% phase B. The flow rate was 0.2 mL/min, the column temperature was 40 °C, and the injection volume was 10 µL. MS data acquisition was performed in both positive and negative modes. The MS parameters were as follows: Ion Source Type = H-ESI; Spray Voltage: Positive Ion (V) = 3200; Spray Voltage: Negative Ion (V) = 2800; Sheath Gas (Arb) = 40; Auxiliary Gas (Arb) = 5. Sweep Gas (Arb) = 0; Ion Transfer Tube Temp (°C) = 350; Vaporizer Temp (°C) = 275; Scan Range (m/z) = 100–1500.

The identification was performed using the Compound Discoverer 3.3 software (ThermoScientific). Only ions with a signal at least three times greater than the background noise and a signal intensity greater than 105 were considered. The annotations obtained were filtered by considering only those with a tolerance of 5 ppm and a cosine greater than 0.8.

### 2.4. Cell Lines

For the evaluation of the cytotoxic and anti-CHIKV activities of *Spirulina maxima*. The following cell lines were employed: VERO (African green monkey kidney cells); Hep G2 (Liver, Carcinoma; Hepatocellular), and BJ cells (Fibroblast, Skin; Foreskin). All cell lines were acquired from the American Type Culture Collection (ATCC; Manassas, VA, USA). Cell culture was carried out in DMEM-F12 medium (12500062, Gibco, Grand Island, NY, USA) supplemented with 10% Fetal Bovine Serum (FBS) and 1% penicillin–streptomycin (L0022, Biowest, Nuaillé, France). Each cell line was cultured at 37 °C in a 5% CO_2_ incubator until it reached 80–90% confluence before being used in experiments.

### 2.5. Virus Stock

The Chikungunya virus employed was obtained as previously described [32]. The strain used in this study was hChik/Mexico/YUC_InDRE_10815/2015, III_Asian genotype, accession No.: (EPI_ISL_19196361, GISAID), isolated from a confirmed sample of a Mexican patient, and subsequently, consecutive passages were performed in Vero E6 cells.

### 2.6. Evaluation of the Cytotoxic Activity of Spirulina maxima (Arthospira) Extracts

To evaluate whether the extracts of this microalga have any cytotoxic effect, the 3-(4,5-dimethyl-2-thiazolyl)-2,5-diphenyl-2H-tetrazolium bromide (MTT) assay (M6494 Invitrogen, Thermofisher, USA) (MTT) was used. The cell lines selected for each assay were seeded in 96-well plates (1 × 10^4^ cells/well). Before the cytotoxicity evaluation, the plate was incubated for 24 h to allow cell adhesion and stabilization. The culture medium was removed from the wells and replaced with fresh culture medium supplemented with different concentrations of the extracts. The cytotoxicity of SP-W was evaluated in VERO cells at concentrations ranging from 25 to 500 µg/mL. In the case of the SP-H, SP-D, and SP-M extracts, these were evaluated at concentrations of 50 and 100 mg/mL in the same cell line. Once it was established that SP-M contained the molecules with the desired activity, its cytotoxicity was evaluated in the three cell lines used in this study (VERO, Hep G2, and BJ) by constructing a dose-response curve ranging from 10 to 100 µg/mL. The treatments were applied for 48 h, as this is the time during which plaques produced by CHIKV can be quantified. After incubation, the medium containing the treatment was discarded. An MTT solution was used at a concentration of 1 mg/mL. A total of 100 µL of this suspension was placed in each well, and the plates were incubated for 1 h at 37 °C in a 5% CO_2_ atmosphere to allow the biotransformation of MTT by viable cells. The MTT solution was discarded, and the formed formazan crystals were dissolved in dimethyl sulfoxide (DMSO). Optical density was measured at 540 nm using a microplate spectrophotometer (Multiskan MCC; Thermo Fisher Scientific, Waltham, MA, USA). The optical density from the control was considered as 100% viability to determine the percentage of viability in the treatments.

### 2.7. Anti-CHIKV Activity

#### 2.7.1. Evaluation of the Extracts

First, the SP-W extract was evaluated to determine whether there were molecules with anti-CHIKV activity in the commercial formula used. VERO cells were seeded in 24-well plates at a density of 1 × 10^5^ cells/well and incubated for 24 h to allow adhesion of cells and monolayer formation. As it was not known whether the molecules of interest had an antiviral or virucidal effect, the viral particles were subjected to treatment before infection. The necessary volumes of DMEM F12 0% medium and CHIKV virus stock were placed in a 1.5 mL microtube to infect the cells at an MOI of 1. The SP-WW extract was added at concentrations of 50, 100, 125, 200, 250, 400, and 500 µg/mL. These treatments were incubated at 37 °C for 1 h with 5% CO_2_ before infecting VERO cells. After incubation, the medium from the wells was discarded, and the treatments with the virus previously exposed to different concentrations of SP-W were placed. Cell infection was carried out for 1 h, and the plate was shaken every 15 min. At the end of this period, the inoculum was removed, and the cells were washed once with 1X PBS to remove unabsorbed virus particles. Then, 2% DMEM F12 medium supplemented with different concentrations of SP-W was added, and the plate was incubated for 24 h at 37 °C with 5% CO_2_. After incubation, the medium was removed, and a covering medium (DMEM F12 with 2% FBS and 1% carboxymethyl cellulose) was added. The plates were incubated at 37 °C with 5% CO_2_ and monitored until plaques appeared. After this, the overlay media was discarded, and the monolayers were washed twice with 500 µL of PBS 1X, and were fixed with 5% formaldehyde for 15 min, and stained with 0.5% crystal violet (Sigma-Aldrich Chemical, St. Louis, MO, USA).

Once the anti-CHIKV activity was demonstrated, the extracts obtained from the powder were evaluated in the same way as SP-W, by incubating the virus with each extract (SP-H, SP-D, and SP-M). at concentrations of 50 and 100 µg/mL for 1 h before infecting the cells. Additionally, a treatment with the same volume of DMSO as the extract was used as a control. This control group was used as 100% PFUs, and the % PFUs in the extracts was determined.

As SP-M demonstrated the most potent anti-CHIKV activity, a dose-response curve was constructed for this extract. The virus was again incubated with each of the different concentrations of SP-M (0, 1.5, 3, 4.5, 6, 7.5, 9, 10.5, 12, 13.5, and 15 µg/mL) for one hour, and then the VERO cells were infected with the inoculum of the CHIKV treated with SP-M.

To demonstrate that the effect of inhibiting virus replication is independent of the cell line used, the test was performed on mammalian cell lines Hep G2 and BJ. The same concentrations as those used in VERO cells were evaluated in these cell lines. Cell infection was conducted as mentioned for the SP-M curve, and after 48 h of incubation, the treatment supernatants were collected and centrifuged at 1500 rpm to remove cell debris. The supernatants were titrated in Vero cells by a plate assay, so 30 microliters of the supernatant were taken and diluted in 270 microliters of DMEM F12 0% to obtain a 1:10 dilution. This was then diluted again with the same volumes of inoculum and medium to obtain a 1:100 dilution, and so on until the dilution in which the plates could be quantified was obtained. From each of these dilutions, an inoculum of 100 microliters was placed on the Vero cell monolayer to perform the infection and quantify the PFU/mL.

#### 2.7.2. Time of Addition Assay

To explore the possible mechanism of action of the molecules responsible for the anti-CHIKV activity, we evaluated the effect of SP-M extract treatment at two different concentrations (7.15 µg/mL (IC50 estimated in BJ cells) and 15 µg/mL (the concentration at which total inhibition is observed)). Antiviral CHIKV activity was assessed at three different time points during infection: before infection (pre-treatment of cells), during infection (co-treatment), and after infection (post-treatment).

For pre-treatment, CHIKV virions that reached 1 MOI were placed in serum-free medium. The medium was supplemented with the evaluated concentrations of SP-M. The mixture was homogenized and incubated for 1 h at 37 °C. The monolayer of BJ cells was infected with a mixture of CHIKV and SP-M for 1 h after the inoculum was removed. The monolayer was washed twice with 1X PBS to discard virus particles that did not penetrate the cells. Fresh medium containing 10% FBS was added, and the plate was incubated for 48 h.

For co-treatment, the inoculum used to infect BJ cells at 1 MOI was added to the necessary volume of SP-M to reach the evaluated concentrations. The inoculum treated with SP-W was used to infect the cells for 1 h. After infection, the inoculum was removed, and the cells were washed with PBS 1X, and fresh medium at 10% SFB and free of SP-M was placed as in the previous case.

In the case of post-treatment, BJ cells were first infected for 1 h, followed by the addition of fresh culture medium without SP-M. Infected cells were treated with different concentrations of SP-M at three different time points (2, 4 and 8 h), after which the culture medium without SP-M was removed and replaced with medium containing different concentrations of SP-M at each time point. In all three cases, the cultures were incubated for 48 h after infection and treatment. The supernatants were subsequently collected and titrated against VERO cells using a plate assay as described in 2.7.1.

BJ cells infected at 1 MOI were used as the control group. They were treated solely with the same volume of vehicle (DMSO). The reduction in virus replication was determined by the percentage of plaque reduction between the methanolic extract of SP concentrations used with respect to that of the control group.

#### 2.7.3. Quantitative Reverse Transcription Polymerase Chain Reaction (RT-PCR)

RT-PCR verified the anti-CHIKV effect, confirming the inhibition of viral genetic material replication. The anti-CHIKV activity was confirmed in VERO, Hep G2, and BJ cell lines using an IC_50_ estimated for every cell line and 15 µg/mL, which was the concentration at which total inhibition was observed. For each of these assays, the cell lines were seeded in 6-well plates at a density of 5 × 10^5^ cells/well and infected at 1 MOI. After infection, the cells were treated with the mentioned concentrations for 48 h. The cells and supernatant were then recovered, and total RNA was extracted using the QIAamp Viral RNA Mini Kit (Cat. No. 52906; QIAGEN, Germantown, MD, USA) according to the manufacturer’s instructions. Total RNA was stored at −80 °C until use.

The CT values obtained from the amplification of the gene encoding the non-structural protein 4 (nsP4) of CHIKV were compared with those obtained in the control group. Amplification was performed in a LightCycler 480 instrument (Roche, Indianapolis, IN, USA) using the primers CHIKV 6856: 5′ TCACTCCCTGTTGGACTTGATAGA 3′ and CHIKV 6981: 5′ TTGACGAACAGAGTTAGGAACATACC 3′, and the fluorogenic TaqMan probe CHIKV 6919-FAM: 5′ AGGTACGCGCTTCAAGTTCGGCG 3′ and a Superscript III Platinum enzyme One-Step RT-qPCR Kit (Cat. No. 1732088; Invitrogen, Thermofisher, Austin, TX, USA). The results were obtained from at least three independent experiments.

### 2.8. Statistical Analysis

Values are expressed as the mean  ±  SD for each study group, in triplicate of three independent assays. Statistical analyses were performed using SPSS Statistical Software version 22.0 for Windows (Chicago, IL, USA). The level of significance was set at *p*  ≤  0.05. One-way analysis of variance (ANOVA) and Tukey’s tests were used to compare the averages between groups. They were conducted to determine the presence of significant differences in the data obtained.

## 3. Results

### 3.1. Cell Viability and Cytotoxicity Assay of Extracts

**Figure 1:**
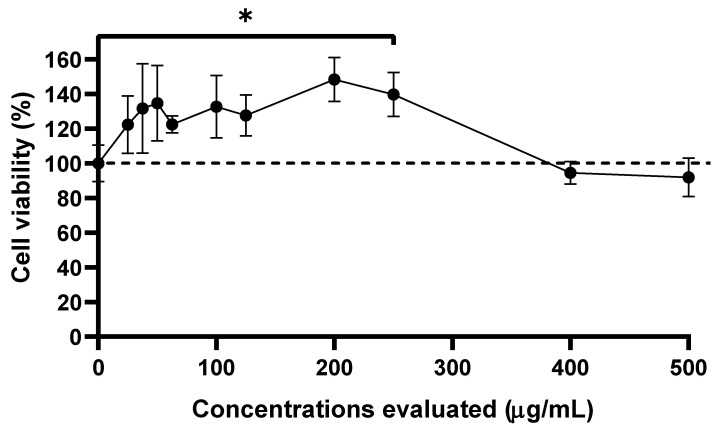
Effect of SP-W on VERO Cell Viability. The results are presented as the percentage of cell viability. Each point represents the mean ± standard deviation observed from the triplicates performed for cell viability. One-way ANOVA and post hoc Tukey’s test. * represents a *p*-value  <  0.05 with respect to the control group.

In the case of SP-W, VERO cell viability remained above 100% at the first concentrations (25 to 250 µg/mL). At higher concentrations (400 and 500 µg/mL), the viability decreased below 100%. However, this decrease was not statistically significant compared to that in the control group, with a CC_50_ estimated at >500 µg/mL for this extract (Figure 1).

The evaluation of the other extracts showed similar results, since none of the extracts showed a decrease below 95% in the viability of VERO cells at any of the concentrations evaluated (50 and 100 µg/mL) (Table 1), demonstrating that the use of this food supplement does not represent a cytotoxic risk in vitro.

**Figure 2:**
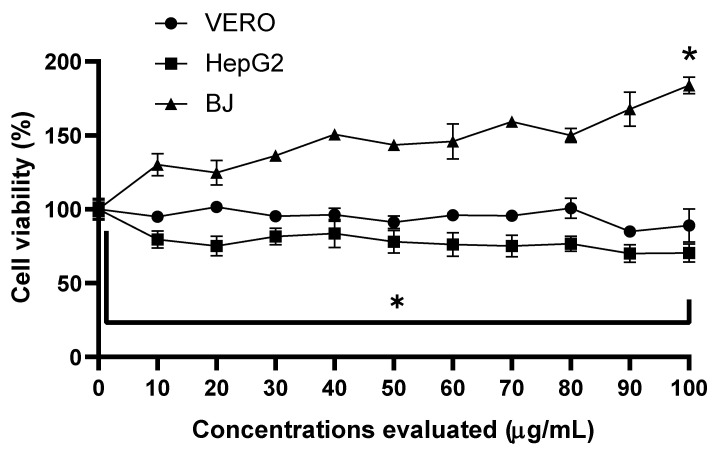
Cytotoxicity of methanolic extract SP in Vero, HEPG2, and BJ cells. Comparison of the percentage of cell viability, as measured by the MTT assay. All data represent three independent experiments performed in triplicate. One-way-ANOVA * *p* value  <  0.05, with respect to the control group, VERO, and HepG2 cells.

The cytotoxicity of the SP-M extract in the three selected cell lines (Figure 2) showed that similar values were obtained in the BJ cell line as those observed in VERO cells. The viability did not decrease significantly with any of the concentrations evaluated, and it was observed to be above 100% at all concentrations used. In Hep G2 cells, a significant (*p* < 0.001) decrease in viability (80.79 ± 9.58%) was observed at the lowest concentration evaluated (10 µg/mL). Although there was a significant decrease in viability, it never fell below 50%, as at the maximum concentration evaluated (100 µg/mL), viability only decreased to 70.43 ± 2.06%. This indicates that the IC_50_ value was above 100 µg/mL (IC_50_ > 100 µg/mL).

### 3.2. Anti-CHIKV Activity of SP-W

**Figure 3:**
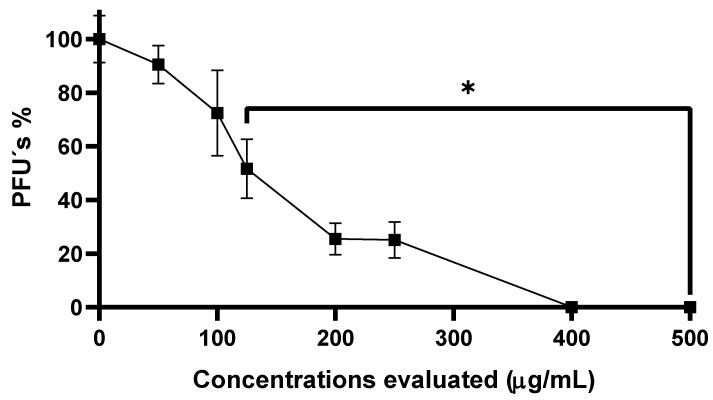
Anti-CHIKV effect of SP-W. The bars represent the ± standard deviation observed from the mean of triplicates for dose-dependent inhibition. One-way analysis of variance (ANOVA) and Tukey’s post hoc test were used to determine the presence of statistical differences. * Represents *p* < 0.05 compared to the control.

Our results demonstrate that the commercial formula of S. maxima contains water-soluble molecules capable of inhibiting Chikungunya virus replication. The decrease in PFU was significant with respect to the control group from a concentration of 125 µg/mL. This implies that the effect on reducing plaque formation is greater as the concentration of SP powder increases, suggesting a dose-dependent effect across the range of concentrations used (50–500 µg/mL). At a concentration of 200 µg/mL, a decrease of more than 50% in the reduction in PFUs was observed, reaching a 26% reduction at this concentration. Furthermore, at the highest concentrations tested (400 and 500 µg/mL), the inhibition of the number of PFUs was complete (100% inhibition), with a significant difference (*p* ≤ 0.01) with respect to the control group (DMEM with 0.1% DMSO was used as vehicle control), (Figure 3).

### 3.3. Evaluation of Anti-CHIKV Activity of Three Extracts Obtained from SP Powder

As shown in Table 2, the SP-H and SP-D extracts showed zero or minimal desired biological activity. The hexane extract failed to significantly reduce the number of PFU’s at either of the two concentrations evaluated. At a concentration of 50 µg/mL, the PFU only decreased by 0.3% with respect to the control group, and at a concentration of 100 µg/mL, the percentage of PFU obtained was above that of the control (104.2%); thus, the hexane extract did not have molecules with the desired activity. The dichloromethane extract significantly reduced the percentage of PFUs at a concentration of 50 µg/mL (89.9%). At a concentration of 100 µg/mL, the rate of PFUs decreased to 55%. Although there was a decrease with this extract, it was not as great as in the case of the SP-M extract; in the latter, the percentage of PFU’s was 0% in the two concentrations evaluated. Therefore, this extract was selected for subsequent tests because it had the most significant desired effect with little or no cytotoxicity.

### 3.4. Anti-CHIKV Activity of Methanolic Extract from SP Powder

**Figure 4:**
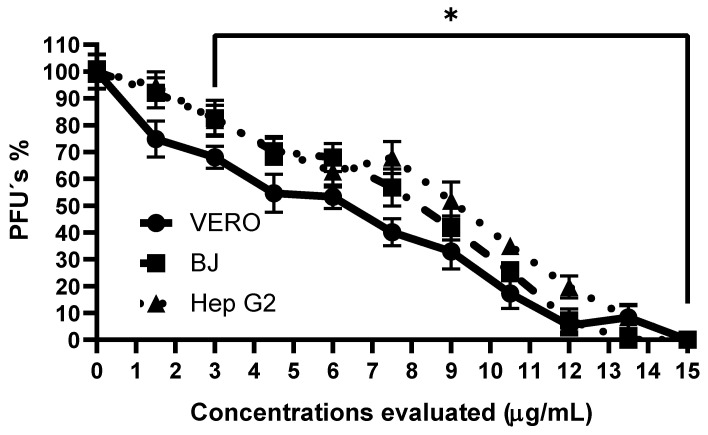
Evaluation of anti-CHIKV activity of SP-M in three cell lines. Each point corresponds to the average of six replicates (two technical and three biological replicates). * Represents a *p*-value < 0.05 with respect to the vehicle control, estimated by one-way ANOVA followed by Dunnett’s test.

As shown in Figure 4, in the case of the VERO and Hep G2 cell lines, CHIKV replication was completely inhibited at 15 µg/mL. In BJ cells, this total inhibition was reached at a concentration of 12 µg/mL. The decrease in the percentage of PFU was significant (*p* < 0.001) from a concentration of 3 µg/mL in the case of the three cell lines. When comparing the IC_50_ values obtained in these (VERO = 6.49 ± 0.637 µg/mL; BJ = 7.15 ± 1.27 µg/mL; Hep G2 = 7.18 ± 1.45 µg/mL), it was observed that there was no significant difference between them (*p* = 0.3996); therefore, the magnitude of the anti-CHIKV effect was independent of cell type.

### 3.5. Addition Time Assay of SP-W

**Figure 5:**
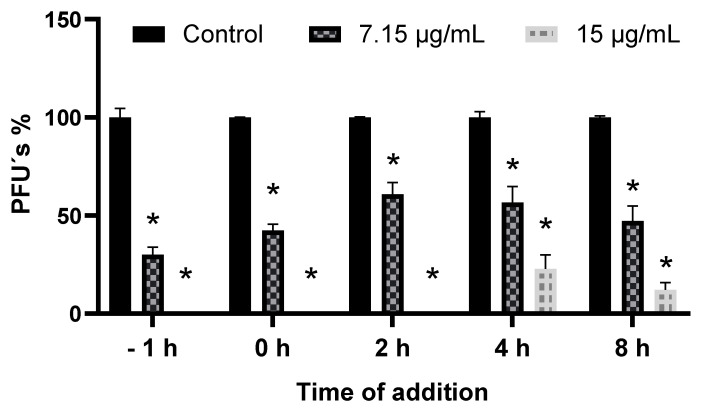
Time of addition assay. Time-of-addition studies were conducted to identify when the SP metabolic extract exerts its antiviral effect during the CHIKV replication cycle. All data are presented as the mean of three independent experiments. One-way ANOVA and Tukey’s post hoc test were used, and * indicates significant differences between groups (*p* < 0.01) with respect to the control.

Figure 5 shows that when using the IC50 estimated in the BJ cell line (7.15 μg/mL), the inhibition in the percentage of PFU’s remains very close to the expected 50% (−1 h = 69.9%; 0 h = 57.6%; 2 h = 39.2%; 4 h = 43.4%, and 8 h = 52.7%). It is important to highlight that at 2 h post-infection, the lowest value was observed, and from this, an apparent increase in the percentage of PFUs was observed. In the case of the concentration of 15 µg/mL it is observed that in the first times (−1, 0 and 2 h) the percentage of inhibition of PFU’s was 100% However in the following times (4 and 8 h) the PFU’s percentage increased to 78.7% and 87.9%, respectively, thus demonstrating that the time in which the treatment is applied has a direct effect on the inhibition of PFU. It is worth mentioning that in all cases, the decrease in the number of PFU’s was significant (*p* < 0.01) with respect to the control group.

### 3.6. Inhibition of Viral Replication by Quantitative Reverse Transcription Polymerase Chain Reaction (qRT-PCR)

**Figure 6:**
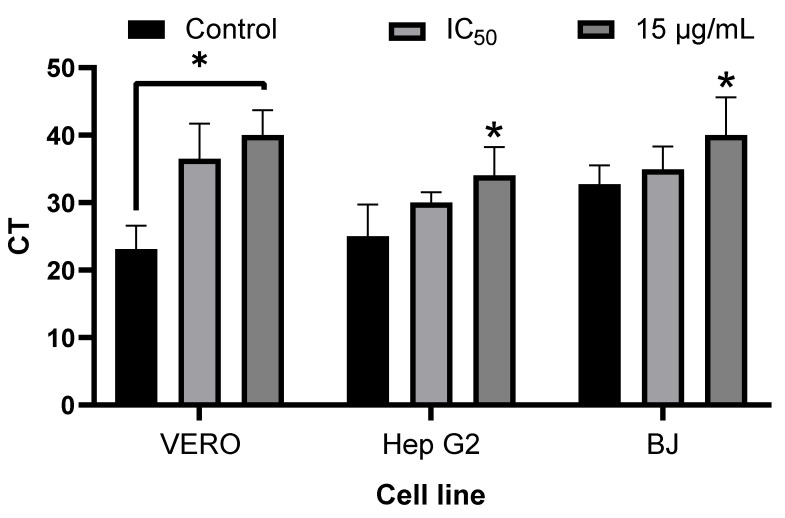
Evaluation of the inhibition of CHIKV viral genome replication by RT-PCR in VERO, HepG2, and BJ cell lines. The IC50 values for the three cell lines were Vero = 6.49 µg/mL, Hep G2 = 7.18 µg/mL, and BJ = 7.5 µg/mL. The differences between the controls and treatments in each cell line were evaluated using 1-way-ANOVA, and Tukey’s post hoc test was used to determine the presence of statistical differences with respect to the control group (* *p* value < 0.05).

As shown in Figure 6, for VERO cells, it is observed that the treatment with the methanolic extract of SP showed a significant difference (*p* < 0.005) in the number of Ct at the two concentrations evaluated (IC_50_ = 6.49 µg/mL and 15 µg/mL) with respect to the control group. In Hep G2 and BJ cells, no significant difference was observed compared to the control group in the IC_50_ concentration estimated for each cell line (7.18 and 7.5 µg/mL, respectively). A significant difference (*p* < 0.005 for the two cell lines) was observed only at a concentration of 15 µg/mL, in which a total reduction in the number of PFUs was observed in the plate tests.

### 3.7. Chemical Composition of SP-M

As shown in Figure S1, chromatograms were obtained in both positive and negative modes, and it was determined that SP-M consists of at least 760 molecules, primarily organic acids, alkaloids, amino acids, carbohydrates, and peptides. However, during filtration, those molecules that did not match the MS/MS spectra in the databases used were discarded. It should be noted that these were the molecules found in the lowest abundance in the sample. At the end of the filtration process, only 197 molecules were considered (Table S1). These were the most abundant (Area (Max.) = 10^10^ to 10^7^) and showed the greatest similarity to the spectra reported in the databases used. Furthermore, of these 300 molecules, the literature research showed that only 21 of them have previously been reported to have antiviral activity, but none of them have been reported to have anti-CHIKV activity.

## 4. Discussion

With respect to the cytotoxic activity of the extracts obtained from the food supplement based on *S. maxima*, it was shown that its use does not represent a risk of cytotoxicity for the cells’ in vitro tests, since none of the extracts obtained at the maximum concentrations evaluated could decrease cell viability. This is because this commercial formula is mainly composed of molecules that can be used as nutrients by the cells, and if this is the case, there is a minimal amount of toxic molecules, as could be the case for the enzyme L-asparaginase, which is present in *S. maxima* cultures [33]. However, this is attributed to its antiproliferative activity against human cancer cell lines, including lung carcinoma A549, hepatocellular carcinoma Hep-G2, and prostate carcinoma PC3. Our results confirmed cytotoxicity in Hep-G2 cancer cells; however, we found that viability did not decrease below 50%. The American National Cancer Institute states that an IC50 ≤ 30 µg/mL is required for an extract to be considered a good candidate for further evaluation as an anticancer agent [34]. Thus, the extract obtained from the commercial formula cannot be considered an anticancer agent. Furthermore, given that we are dealing with cancer cells, we cannot guarantee that this extract will have a hepatotoxic effect on healthy cells, and this risk would be significantly reduced by isolating only the molecule responsible for the anti-CHIKV effect.

Furthermore, although it has been reported that molecules such as water-soluble polysaccharides can decrease the viability of normal human cells by 19.1% [35], these are obtained by ultrasonic extraction, which allows obtaining these polysaccharides in a relatively intact manner, which does not occur with the extraction method used in the present study, thus decreasing the potential of these extracts as anti-carcinogenic but allowing obtaining extracts with good anti-CHIKV activity, highlighting the importance of the different extraction methods for the desired biological activity.

Once the SP-M extract was selected as the one with the greatest anti-CHIKV activity, its cytotoxicity was evaluated in three different cell lines, VERO, Hep G2, and BJ, to establish whether there was any difference in the cytotoxic and anti-CHIKV effects of the molecules present in the extract. Our results show that the SP-M extract does not contain molecules with cytotoxic activity, at least not at the maximum concentration evaluated for these assays (100 µg/mL). This phenomenon was observed in the three cell lines used, despite their origin from two different organisms, *Cercopithecus aethiops* and *Homo sapiens*. However, we can appreciate a different behavior between the cell lines of humans, since in the case of BJ cells, the percentage of viability increases significantly when the concentration increases, whereas in Hep G2 cells, viability remains below 100%. One possible cause of this behavior is that, despite being human cells, both cell lines represent different cell types. The Hep G2 line consists of epithelial cells. At the same time, BJ are fibroblasts; this difference can be translated into morphological and metabolic differences between the cells. For example, the morphology of mitochondria differs between cell and tissue types, and this difference has significant repercussions at the metabolic level [36]. In addition, even occurrences between cell lines from the same tissue, as is the case with liver tissue, in which the expression profile of drug-metabolizing enzymes and transporters varies significantly between liver cell lines and primary hepatocytes [37]. Another significant difference between these human cell lines is that the HepG2 cell line corresponds to epithelial cells based on its morphology, whereas the BJ cell line is a fibroblast. Our results suggest that some molecules in SP-M may play a role in wound healing, given that fibroblasts are responsible for repairing the extracellular matrix [38].

Regarding the antiviral activity of the molecules present in SP, the results show that when extracted with water (SP-W), molecules with anti-CHIKV activity can be obtained in the commercial formula. From this, we can infer that consumption of this could help in the prevention and/or treatment of chikungunya virus; however, this should be analyzed further in future research, as there may be low absorption of the molecules responsible for the activity when administered orally [39]. This is the case for amprenavir, an HIV-1 protease inhibitor, which has already been shown to have first-pass intestinal metabolism that drastically decreases (approximately 10 times) the absorption of this molecule when administered orally [40]. Another possibility is that the molecules undergo a drastic first-pass effect, modifying their structure in such a way that they lose their activity or, worse, result in toxic metabolites [41]. Our results show that an anti-CHIKV effect was observed. However, it was observed at very high concentrations; therefore, it is assumed that the molecules responsible for this effect are present at very low concentrations, which could result in zero or low activity when consumed in this manner. Therefore, we decided to increase the concentration of these molecules by maceration with solvents of different polarities.

Since SP-M showed no cytotoxic effects and a significant anti-CHIKV effect, this extract was selected for further evaluation. Therefore, a dose-response curve was generated using this extract in the three cell lines selected in this study. The first cell line selected was VERO, which is commonly used in studies involving viruses because it is easy to handle and maintain, and it can adapt to a culture medium with a low or even zero amount of serum. This allows different protocols to be implemented in the management of viruses [42]. In addition, another essential characteristic is its deficiency in the secretion of the signal peptide interferon when infected by a virus, which results in a deficient expression of interferon, making these cells permissive to the infection of a large number of viruses from groups I, III, IV, and V [43,44]. The second cell line selected was HepG2, a *Homo sapiens* cell line, which can provide a better picture of what could happen in human infection. In addition, these epithelial cells have already been shown to be prone to CHIKV infection [45,46] since it has been shown that this virus has tropism for various cells in the body. In addition, epithelial cells are among the first cells to come into contact with the virus during infection [47]. Finally, the BJ cell line was selected for three reasons: first, this line corresponds to human fibroblasts, which are considered the main site of replication of the CHIKV [48]; second, it corresponds to the fact that these cells come from normal tissue, unlike HepG2 cells, which come from carcinoma. Finally, these types of cells are involved in the host immune response through the production of tumor necrosis factor (TNF), interleukin 6 (IL-6), and cytokines in response to any infection with enveloped RNA viruses transmitted by arthropods, as is the case with CHIKV [49]. Our results demonstrate that the anti-CHIKV activity of the molecules present in SP-M is independent of the cell line used, as the intensity of the effect was similar, and no significant differences were observed between the values obtained or the IC_50_ determined for these three lines. This suggests that the mechanism of action may have an antiviral effect, as it directly interferes with one or more processes of viral replication. The addition time test supports this hypothesis. The results obtained demonstrate that when using the estimated concentration of the IC_50_ (7.15 µg/mL), the percentage of PFU’s was lower than 50% at 2 hpi (39.2%), which means that in this period, the viral replication process is more affected by the molecules present in SP-M. At this time, transcription of the viral genome mediated by the machinery of the host cell begins, giving rise to the four non-structural proteins of the virus to start the process of generating the early replication complex [50]. Therefore, by observing that the decrease in the number of PFU’s is more marked at this time, we can infer that the mechanism of action of this extract is directed to one or more of the proteins responsible for the translation of the viral genome, thus demonstrating an antiviral effect of these molecules. We also observed a similar phenomenon at a concentration of 15 µg/mL, where at 4 hpi, the inhibition of viral replication was not 100% because that period of time it is possible that in some of the cells, the process of translation of the viral genome had already begun, which explains why the inhibition at that time was not total.

Finally, to verify the anti-CHIKV effect observed in the plaque assays, viral RNA detection was performed using RT-PCR. This method was chosen because it is a widely used technique for the diagnosis of CHIKV. It has been shown that this can indicate a decrease in the replication of CHIKV’s genetic material when evaluating molecules with this activity [51,52]. It was observed that, at the IC_50_ calculated for each cell line, only VERO cells showed a statistically significant difference with respect to the control group. However, in the other two cell lines (Hep G2 and BJ), although the difference was not significant, it was similar to that observed at the highest concentration. Since the difference was significant in the three cell lines, this confirms that SP-M contains molecules capable of inhibiting CHIKV replication. When evaluating the concentration at which total inhibition of PFU’s was observed in the plate assay (15 µg/mL), it was still possible to detect the presence of viral RNA in these samples. This may be because the technique is more sensitive and focuses on amplifying a sequence of a non-structural protein (nsP4). The detection of these low amounts of RNA could be due to the fact that some molecules responsible for the desired activity might require activation by the metabolism of the cells through some modification, such as phosphorylation, as is the case with ribavirin [53]. This would allow the replication of the virus’s genetic material to begin, reaching sufficient concentration of the active form of the molecule, which explains the detection of the viral RNA. Furthermore, it is also possible that the activity of the molecules present in SP-M decreases the content of some molecules essential for the replication of the virus, such as polyamines, which have already been shown to be essential in the replication of CHIKV and whose production can be inhibited by N-ω-chloroacetyl-L-ornithine, a competitive inhibitor of ornithine decarboxylase [54]. In this case, the concentration in the extract may not be high enough to have this effect immediately, allowing the start of viral RNA replication but inhibiting this process as the effect accumulates.

The low amount of RNA that can be detected by this technique also confirms that the mechanism of action is directly related to the inhibition of viral RNA replication. If the inhibition was related to viral entry, it would not be possible to detect viral RNA because washes were performed to discard particles from the initial infection inoculum. If the mechanism of action is related to viral assembly and maturation, it would be possible to detect a greater amount of viral RNA, as its replication would not be affected. This is similar to the effect of the steroidal alkaloid stomatidine, present in green tomatoes, which can inhibit the release of CHIKV particles but not the replication of viral RNA, allowing it to accumulate within the host cell.

As the results show, the mechanism of action is involved in the early stages of viral replication. As a prospective approach to defining the mechanism of action of the molecule(s) responsible for anti-CHIKV activity, a drug affinity responsive target stability (DARTS) assay can be performed with protein extracts from infected cells and the isolated molecules responsible for the activity. This will allow us to observe the compound-protein interaction and thus definitively determine whether it involves the inhibition of viral uptake, the entry process, or the inhibition of one of the virus’s non-structural proteins [55,56].

Regarding the phytochemical composition of SP-M, the LC-MS/MS analysis revealed that SP-M is a complex mixture composed of at least 560 compounds, including organic acids, fatty acids, aliphatic alcohols, alkaloids, amino acids, carbohydrates, flavonoids, lipids, peptides, polyketides, phenyl propanoids, terpenoids and vitamins, which is consistent with the literature [24]. Since it is such a complex mixture, it would be difficult to assure which of these molecules are responsible for the anti-CHIKV activity. However, in the filtrate of compounds that we carried out, octadecadienoic acid derivatives are found in large proportion (9-Oxo-10(E),12(E)-octadecadienoic acid; (9Z,12Z)-6,8-Dihydroxy-9,12-octadecadienoic acid and (9Z,12Z)-6,8-Dihydroxy-9,12-octadecadienoic acid), and the molecule 13S-Hydroxy-9Z,11E,15Z-octadecadienoic acid is found. These molecules have already been reported to have activity against the Rift Valley Fever Virus, which is also an arbovirus that can be transmitted by mosquitoes of the genus Aedes [57], it is possible that these molecules may also have anti-CHIKV activity. In this same sense, among the molecules found in highest proportion in SP-M, there is Azelaic acid, a saturated dicarboxylic acid which has been reported to be able to inhibit the dengue virus, another arbovirus transmitted by mosquitoes of the genus Aedes, by 36.6% at a concentration of 1 mM [58]. Another molecule found in SP-M is Betaine, which has been shown to have activity against hepatitis B and dengue viruses [59,60]. Similarly, we found several molecules that have been reported to have antiviral activity; however, none of the molecules found in the analysis have been reported to have anti-CHIKV activity. Therefore, it would be advisable to make the effort to isolate the molecule or molecules responsible for this activity through a bio-directed assay, first by fractionating this extract using column chromatography until a much less complex mixture is obtained, from which preparative thin-layer chromatography (prep TLC) or preparative HPLC could be performed to obtain the pure compounds and evaluate the anti-CHIKV activity either separately or together in case to evaluate any synergistic effect and finally perform its characterization and elucidation of its structure using Magnetic nuclear resonance (NMR).

## 5. Conclusions

This report presents the first demonstration of the anti-CHIKV activity of molecules present in *Spirulina maxima*, which exhibits strong anti-CHIKV potential. Notably, these molecules did not exhibit cytotoxic effects at high concentrations but demonstrated potent anti-CHIKV effects, completely inhibiting viral replication at low concentrations. Therefore, future research should focus on purifying and identifying the molecules responsible for the desired effects. Furthermore, our results demonstrate that the molecules present in SP-M can interfere with or inhibit some of the processes related to the early stages of infection, such as the entry of the virus or the first steps of viral genome replication, exhibiting an antiviral mechanism of action.

## Figures and Tables

**Table 1:** Cytotoxic effects of SP extracts.

Concentration (µg/mL)	Cell Viability (%) ± SEM		
	SP-H	SP-D	SP-M
50	96.4 ± 5.37	98.6 ± 7.84	106.7 ± 11.4
100	99.8 ± 6.72	97.3 ± 8.34	96.49 ± 8.9

**Table 2:** Anti-CHIKV effects of three extracts obtained from SP powder.

Concentration (µg/mL)	% PFU’s ± SEM		
	SP-H	SP-D	SP-M
50	99.78 ± 7.61	89.91 ± 7.91	0
100	104.2 ± 5.77	55.04 ± 14.47 *	0

* Represents 0. compared to the vehicle control.

## Data Availability

The data supporting the findings of this study are available from the corresponding author upon reasonable request.

## Supplementary Materials

The following supporting information can be downloaded at: https://www.mdpi.com/article/10.3390/v17121583/s1, Figure S1. Total Ion Chromatogram (TIC) of SP-M in both positive and negative modes; Table S1. Molecules present in SP-M.
