# Supplementary material for: In Vitro Evaluation of the Antiviral Effect of Spirulina maxima (Arthrospira) Alga Against Chikungunya Virus
Source: Viruses. 2025 Dec 5;17(12):1583. doi: 10.3390/v17121583 (PMC12737399; doi:10.3390/v17121583)
Supplement: Supplementary file 1 [file viruses-17-01583-s001.zip › viruses-3964130-supplementary.docx]

Figure S1. Total Ion Chromatogram (TIC) of SP-M in both positive and negative modes

Table S1. Molecules present in SP-M

The compounds marked in green are those that have been reported to have antiviral activity.

Table S1. Molecules present in SP-M

The compounds marked in green are those that have been reported to have antiviral activity.

Table S1. Molecules present in SP-M

The compounds marked in green are those that have been reported to have antiviral activity.

Table S1. Molecules present in SP-M

The compounds marked in green are those that have been reported to have antiviral activity.
